# Retraction Note: The effects of a recruitment manoeuvre with positive end-expiratory pressure on lung compliance in patients undergoing robot-assisted laparoscopic radical prostatectomy

**DOI:** 10.1007/s10877-023-00993-8

**Published:** 2023-03-13

**Authors:** Osamu Kudoh, Daizoh Satoh, Naosuke Hori, Izumi Kawagoe, Eiichi Inada

**Affiliations:** Department of Anesthesiology and Pain Medicine, Juntendo University School of Medicine, 2-1-1 Hongo, Bunkyo-ku, 113-8421 Tokyo, Japan

**Retraction Note: Journal of Clinical Monitoring and Computing (2019) 34:303–310**

10.1007/s10877-019-00306-y

The Editor in Chief has retracted this article. After an investigation by Juntendo University Hospital it was discovered that the authors made changes to the study protocol without obtaining ethical approval. Osamu Kudoh has stated on behalf of all co-authors that they do not agree to this retraction.

